# Oral Sensitivity to Flowability and Food Neophobia Drive Food Preferences and Choice

**DOI:** 10.3390/foods10051024

**Published:** 2021-05-08

**Authors:** Sharon Puleo, Paolo Masi, Silvana Cavella, Rossella Di Monaco

**Affiliations:** 1 Center of Food Innovation and Development in the Food Industry, University of Naples Federico II, 80055 Portici, Italy; pmasi@unina.it (P.M.); cavella@unina.it (S.C.); dimonaco@unina.it (R.D.M.); 2 Department of Agricultural Sciences, Food Science and Technology Division, University of Naples Federico II, 80055 Portici, Italy

**Keywords:** texture sensitivity, food liking, psychological traits

## Abstract

The study aimed to investigate the role of sensitivity to flowability on food liking and choice, the relationship between sensitivity to flowability and food neophobia, and its role in food liking. Five chocolate creams were prepared with different levels of flowability, and rheological measurements were performed to characterise them. One hundred seventy-six subjects filled in the Food Neophobia Scale and a food choice questionnaire (FCq). The FCq was developed to evaluate preferences within a pair of food items similar in flavour but different in texture. Secondly, the subjects evaluated their liking for creams (labelled affective magnitude (LAM) scale) and the flowability intensity (generalised labelled magnitude (gLM) scale). The subjects were clustered into three groups of sensitivity and two groups of choice preference. The effect of individual flowability sensitivity on food choice was investigated. Finally, the subjects were clustered into two groups according to their food neophobia level. The sensitivity to flowability significantly affected the liking of chocolate creams and the solid food choice. The liking of chocolate creams was also affected by the individual level of neophobia (*p* = 0.01), which, in turn, was not correlated to flowability sensitivity. These results confirm that texture sensitivity and food neophobia affect what a person likes and drives what a person chooses to eat.

## 1. Introduction

Food texture plays a pivotal role in how foods and beverages are perceived [1,2] and whether food is liked or disliked [3]. While research has largely explored how individual differences in genetics and physiology, related to taste and odour perception, interact with food experiences to contribute to food likes and dislikes [4,5,6,7], few studies have examined texture perception sensitivity and how it affects food preferences, despite the belief of its importance in food choice [8]. As with the other senses, individual differences in how textures are perceived may contribute to how texture preferences develop.

Despite the awareness that texture influences food acceptance during life [9,10,11], it is doubtful how different texture sensitivities may lead to different food preferences. The approaches proposed across the years do not investigate specific texture attributes but rather the overall (oral/non-oral) tactile acuity, using tools that do not reflect the real perception of the food texture [12,13,14,15,16]. The reason why no study has used real food products to investigate texture sensitivity is the fact that texture is a multi-parameter attribute, related to the structure of foods and detected by several senses [8], and when it is modified in different levels of intensity, the other properties of food (taste, flavour and odour) are differently perceived, as they are also affected by the structure of the food [17]. Therefore, the main trouble related to the use of a real food product is the difficulty in modifying the texture intensity levels of the product without changing any other sensory property. The modification of other sensory properties would influence the sensory perception, and consequently, it could affect food preferences. 

Aware of the gap related to the methods to measure texture sensitivity and of the main troubles affecting the use of real food products, Puleo and colleagues proposed a new approach to measure graininess sensitivity using five samples of chocolate creams, changing only their graininess levels [18]. The authors were able to cluster subjects into three different groups of sensitivity and also found an effect on individual likings; however, they did not investigate whether individual sensitivities could drive the food choice. In addition, they did not explore any possible existing relationships between individual sensitivities and other individual variables, such as psychological behaviour, which, in turn, has been demonstrated to have an important influence on texture perception and liking [19].

The relationships between several psychological domains and different sensory properties have been largely investigated [20,21,22,23]. In particular, in the past two decades, food neophobia, referring to the reluctance to try unknown foods, with genetic and environmental determinants [24,25], has been extensively investigated by taking into account several different personal factors from food preferences to food choice [26,27], from active chemosensory exploration of the world (sniffing and tasting) [22,28] to physiological responses associated with alertness [29]. Spinelli and colleagues found a significant effect of the food neophobia level and perception of burning sensation and acid taste [22]. They observed that more neophobic individuals score the intensity of pungency and acid taste as higher. Thus, this personality trait was associated with a different perception of the key sensation. 

Regarding texture, the enjoyment of different textures is related to food neophobia in young children [30,31] and to picky eating in adults [19]. However, quite surprisingly, there has been little research carried out to ascertain whether texture responsiveness varies according to the degree of food neophobia and whether individual differences in perception may contribute to influencing food preferences and choices among neophobic and neophilic subjects.

With these considerations, the aim of this study was threefold. The first aim was to use the method proposed by Puleo and colleagues [18,19,20,21,22,23,24,25,26,27,28,29,30,31,32] to measure the sensitivity to flowability using chocolate creams as the target real food. Flowability was chosen as a key texture attribute because it is considered one of the most dominant sensory characteristics of semisolid foods [11], together with viscosity. Over the years, several studies have investigated the viscosity perception, and different methods to state individual sensitivities have been proposed [16,33,34]. However, although the authors were able to measure the individual sensitivity to viscosity, the results cannot be generalised, because they were obtained using different methods; therefore, it is not possible to draw a unique conclusion. In addition, to the best of our knowledge, no studies have been conducted on the relationship between flowability sensitivity and food liking and choice. Therefore, the second aim was to verify whether and how different levels of flowability sensitivity could affect food liking and choice.

Finally, the third aim was to analyse the correlation between the sensitivity to flowability and the food neophobia trait and to also investigate the role of food neophobia in food liking.

## 2. Materials and Methods

### 2.1. Sample Preparation

Chocolate mix powder (Paneangeli, Cameo S.p.A., Brescia, Italy) and completely skimmed milk (Berna, Parmalat S.p.A., Milan, Italy) were purchased from a local supermarket and used to prepare samples differing in flowability. Chocolate powder mix and skimmed milk were mixed using an electric whisk, at room temperature, for 2 min, until a homogenous mix was obtained. A panel of 10 assessors selected for general sensory acuity (six females, average age = 23 years) was involved in group discussion evaluations (10 h) to select chocolate creams that differed in flowability but not other properties (taste, flavour, colour) (preliminary results data not reported for the sake of brevity). Eight samples differing in solid concentrations were selected (C0, 29%; C1, 31%; C2, 33%; C3, 36%; C4, 39%; C5, 43%; C6, 48%; C7, 54% (*w*/*v*)). Among them, the C3 concentration (36%) was the one suggested by the company to obtain the optimal and traditional cream. Consequently, the selected assessors performed a ranking test (three replications), during which they were asked to rank the samples from the most to the least flowing one. According to the ranking test results (Supplementary Materials, Table S1), five samples were selected. In particular, only samples C1–C5 were selected since they represented different flowabilities but were not easily recognisable by all the selected judges.

Samples were prepared the day before the sensory test and stored in glass containers at refrigerated temperature (4 °C). Before the sensory test, the samples were equilibrated at room temperature for 2 h.

### 2.2. Rheological Properties: Stress Overshoot

The rheological properties of the samples were determined by a Modular Advanced Rheometer System (Haake MARS, Thermo Scientific, Waltham, MA, USA), equipped with a vane tool geometry (diameter = 22 mm, length = 16 mm, distance = 8.5 mm). Transient tests were carried out, and to this end, the stress (τ, Pa) was measured as a function of time (60 s), keeping the shear rate constant (ɣ = 10 s^−1^). The flow curves were carried out at 30.5 °C, as an arithmetic average of room and mouth temperatures, according to the method proposed by [35]. Three replications for each sample were performed. Results were used to produce a shear stress growth function and to collect stress overshoot values [36].

### 2.3. Consumer Evaluation Overview

The consumer evaluation consisted of two steps. Firstly, at the time of recruitment, participants were requested to complete an online questionnaire, where age and gender were collected, together with the responses to the Food Neophobia Scale and the food choice questionnaire. Secondly, participants were asked to attend one consumer session in individual booths to evaluate the liking and perceived flowability of the five samples of chocolate creams. Further details are explained below.

#### 2.3.1. Participants

A total of 176 Italian subjects (females = 118, age range = 18–70 years old, median age = 25 years) were recruited using social media, flyers and emails (from pre-existing databases) by promoting the study offering a final reward (shopping voucher). Participants signed two copies of written informed consent according to the principles of the Declaration of Helsinki (1964 and its later amendments) and the ethical standards of the University of Naples Federico II. Pregnant women and people with dental/oral issues (e.g., missing teeth, dental prosthesis, braces) were excluded from the study.

#### 2.3.2. Online Questionnaire

Participants were asked to fill in an online survey (Google form) at home at the time of recruitment.

The trait of food neophobia was measured using the Food Neophobia Scale (FNS) developed by [37]. 

The food choice questionnaire was developed to evaluate preferences within a pair (similar in flavour but especially different in texture) of items developed on the basis of texture dichotomies belonging to two texture domains: a liquid texture domain, containing 5 pairs of items, differing in flowability (thin/thick), and a solid texture domain, containing 4 pairs of items, differing in hardness (soft/hard). In Table 1, all the pairs are shown. We started with the hypotheses that going from the thin (liquid domain) to the hard (solid domain) option, four different levels of flowability in the mouth are obtained.

For each pair, respondents were asked to indicate which food they would choose in a normal eating situation, without diet restrictions.

The presentation order of the food items, within and between each pair, was randomised across participants [21,38]. 

#### 2.3.3. Sensory Evaluation

Subjects evaluated the five chocolate creams and scored their liking by using the labelled affective magnitude (LAM) scale, a 100 mm vertical line from 0 (greatest imaginable dislike) to 100 (greatest imaginable like) and with anchor words spaced according to the spacing provided by [39]. In addition, subjects were asked to score the perceived flowability intensity by using the generalised labelled magnitude (gLM) scale, a 100 mm vertical line from 0 (no sensation) to 100 (the strongest imaginable sensation of any kind) and intermediate anchors, as provided by [40]. The subjects were instructed to use the gLM scale following a published procedure [21]. 

Five samples were served to the subjects on plastic teaspoons, in a monadic, randomised and balanced order, identified by three-digit random codes. 

The subjects were asked to use a common procedure to evaluate the flowability intensity, consisting of applying shearing with the tongue against the palate, for a few seconds, when the subjects felt that a judgement could be made [41]. 

The subjects were provided with a cup of still water to rinse their mouth before testing the next sample. Data were collected using Fizz Acquisition software (Biosystèmes, Couternon, France).

### 2.4. Data Analysis

**Figure 1:**
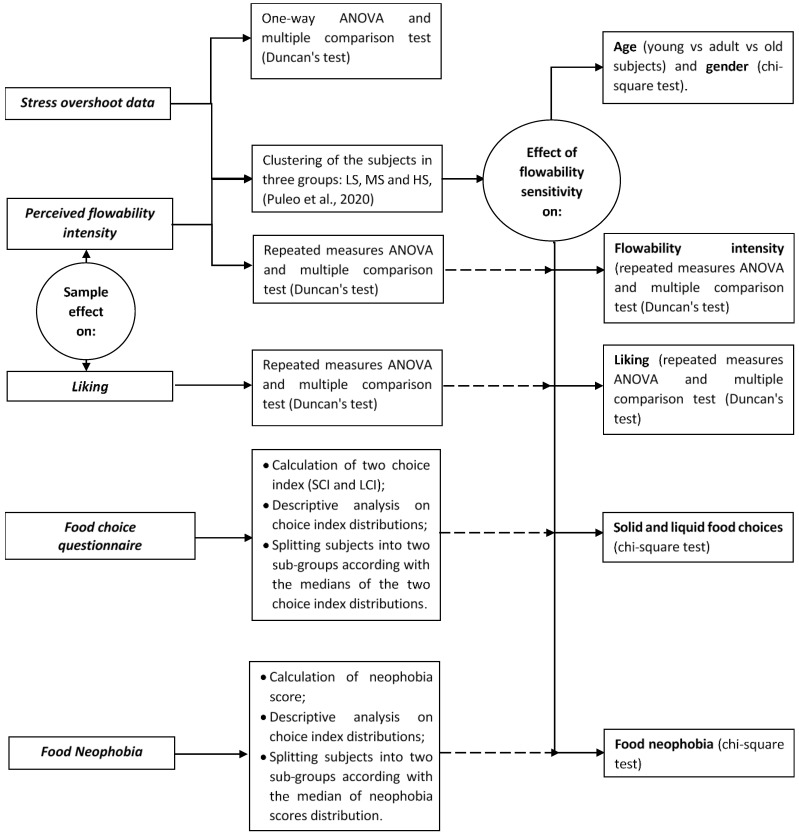
Flowchart of the data analysis.

The data analysis approach is described in (Figure 1) and also detailed below. 

The values of stress overshoot were analysed by means of one-way analysis of variance (ANOVA), and a multiple comparison test (Duncan’s test) was used to statistically compare the samples (*p* ≤ 0.05). 

Prior to analysing sensory data, a normality test was run on the datasets related to perceived flowability intensity, food liking and food neophobia. All the tested datasets followed a normal distribution (Shapiro–Wilk test, *p* > 0.05). 

Repeated-measures ANOVA and a multiple comparison test (Duncan’s test) were used to evaluate whether differences among the samples (used as a repeated factor) were statistically significant (*p* ≤ 0.05) in terms of perceived flowability. At the same time, subjects were split into three groups, lowly sensitive (LS), moderately sensitive (MS) and highly sensitive (HS), according to the approach proposed by Puleo et al. [18,19,20,21,22,23,24,25,26,27,28,29,30,31,32], considering that HS subjects have a discrimination ability comparable to the instrument.

Repeated-measures ANOVA and a multiple comparison test (Duncan’s test) were also used to evaluate whether differences among the samples (used as a repeated factor) were statistically significant (*p* ≤ 0.05) in terms of food liking.

From the food choice questionnaire, a value of 1 was assigned to the thick and hard options, while a value of 0 was assigned to the thin and soft options. Next, a choice index (solid choice index (SCI), liquid choice index (LCI)) for each domain was calculated as a sum of the choice of the thick options and the hard options, with higher scores reflecting a higher choice for the thick and hard options. For each domain, based on the calculated CIs, subjects were split into two sub-groups representing low (thin-food lovers and soft-food lovers, for the liquid and the solid domain, respectively) and high (thick-food lovers and hard-food lovers, for the liquid and the solid domain, respectively) scores, using the median values as a cut-off. Participants with the median score were excluded from the dataset (30 subjects).

From the food neophobia questionnaire, individual FN scores were computed as the sum of ratings given to the 10 statements after the neophilic items had been reversed [37]. Based on the calculated total score, subjects were split into two sub-groups representing low and high scores, using the median value as a cut-off. Participants with the median score were excluded from the dataset (30 subjects) [42].

Next, a chi-square test was run to analyse the relationship between flowability sensitivity (three groups; see above) and age groups and gender.

Thereafter, repeated-measures ANOVA was used to verify the effect of the flowability sensitivity on both perceived flowability intensity and sensory liking.

Moreover, the chi-square test was used to determine the relationship between flowability sensitivity, solid and liquid food choices and food neophobia. 

Finally, repeated-measures ANOVA was used to verify the effect of the two neophobia levels on sensory liking as well.

The XLSTAT statistical software package version 2016.02 (Addinsoft) was used for data analysis.

## 3. Results

### 3.1. Rheological Properties: Stress Overshoot

**Figure 2:**
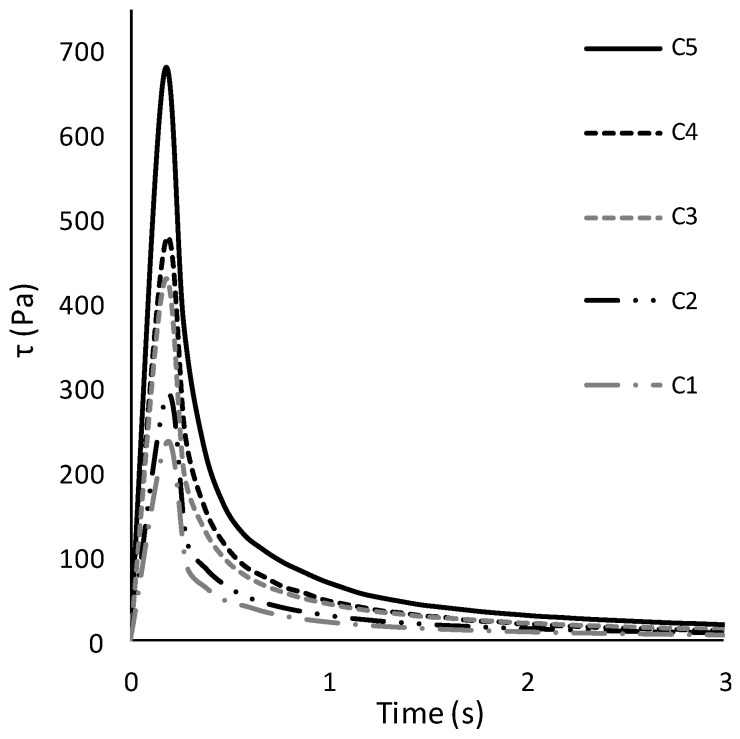
Stress overshoot curves (average of three replications) of chocolate creams differing in solid concentrations (C1, 31%, C2, 33%, C3, 36%, C4, 39%, C5, 43%, (*w*/*v*)).

Shear stress was measured as a function of time at the estimated shear rate of the mouth, according to [29]. Shear stress displays an initial overshoot, at short times, before reaching a steady-state value at long enough times; hence, the phenomenon is commonly referred to as stress overshoot. Figure 2 shows the stress overshoot average curves for each tested sample.

As expected, by increasing the solid concentration, the maximum shear stress value (stress overshoot, Pa) increased accordingly. In addition, the values of the stress overshoot of each sample were extrapolated from each curve and reported as the representative parameter of the flowability of the tested creams. The trend in the stress overshoot was described with a linear equation (*R*^2^ = 0.98). The stress overshoot values were significantly different (Duncan’s test, *p* < 0.0001). 

### 3.2. Sensory Evaluation

A total of 176 subjects scored the food liking and the flowability intensity by using the LAM and gLM scales, respectively. By averaging both food liking and perceived flowability scores, significant differences were found among the samples, as shown in Table 2.

Regarding food liking scores, subjects moved on the scale between the labels *slightly liked* (score ≃ 59) and *liked* (score ≃ 65), with higher values reflecting intermediate concentrations.

Regarding the perceived flowability intensity, subjects moved on the scale around *moderate* and *strong* (score ≃ 16 and ≃33, respectively) and between *strong* and *very strong* (score ≃ 33 and ≃50, respectively), significantly discriminating between the evaluated samples. By looking at the results (Table 2), subjects equally perceived the flowability of the first two samples (C1, 31%; C2, 33% (*w*/*v*)) as well as of the last two (C4, 39%; C5, 43% (*w*/*v*)). The flowability of the intermediate sample (C3, 36% (*w*/*v*)) was perceived as significantly different compared to the perceived flowability of the other samples. 

To cluster the subjects according to their flowability sensitivity, the method proposed by Puleo et al. [18,19,20,21,22,23,24,25,26,27,28,29,30,31,32] was used. Therefore, it was assumed that sensory scores should follow the same trend exhibited by the instrumentally measured stress overshoot, with the increase in the solid concentration, that is, an upward linear relationship. Thus, the flowability scores of each subject were fitted with a linear equation, according to Puleo and colleagues [18,19,20,21,22,23,24,25,26,27,28,29,30,31,32], estimating both the slope and the *R*^2^ coefficient and using them as clustering parameters according to the quartile distribution (Table 3). 

Therefore, subjects whose perceived flowability scores correlated with a linear equation with both a high *R*^2^ coefficient and high slope (values greater than the third quartile, 75% of the distribution) were clustered into the high-sensitivity group (*n* = 45). Accordingly, subjects whose perceived flowability scores correlated with a linear equation with both a low *R*^2^ coefficient and low slope (values lower than the first quartile, 25% of the distribution) were clustered into the low-sensitivity group (*n* = 48). The remaining subjects were clustered in the moderate-sensitivity group (*n* = 83).

Firstly, no significant relationship between flowability sensitivity and age (*p* = 0.297) and gender (*p* = 0.78) was found. Therefore, data were analysed without considering any interaction between these variables.

The scores given by each sensitivity group were submitted to repeated-measures ANOVA, and the significant differences between and within groups were estimated (multiple comparison test, Duncan’s test). In Table 4, food liking and perceived flowability scored by different groups are shown.

As it can be observed in (Table 4), regarding the differences in perceived flowability between the groups, the lowly sensitive subjects evaluated the first two samples with significantly higher scores compared to the moderately and highly sensitive subjects. On the contrary, the lowly sensitive subjects evaluated the last two samples with significantly lower scores compared to the moderately and highly sensitive subjects. 

In addition, regarding food liking, by looking at the differences within each group, lowly sensitive subjects equally liked the five samples, while significant differences were observed within the moderately and highly sensitive subjects. In particular, the most preferred sample by the highly sensitive subjects was the third one (36%, *w*/*v*). 

By looking at the differences between the groups, the flowability sensitivity affected the food liking of only this sample (36%, *w*/*v*). In particular, highly sensitive subjects evaluated this sample with higher scores compared to lowly and moderately sensitive subjects. 

**Figure 3:**
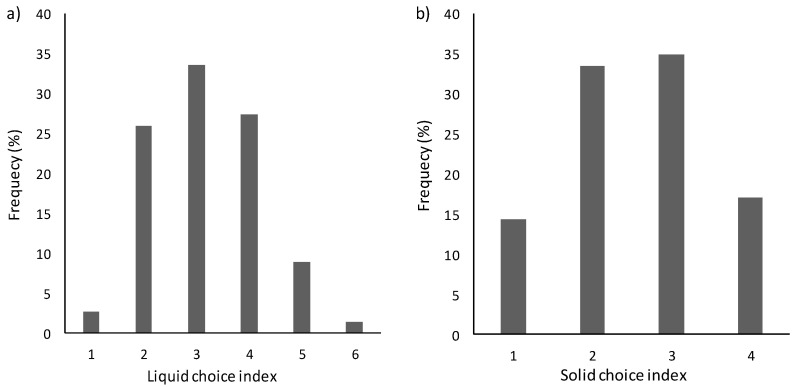
Choice index for the liquid domain (**a**) and the solid domain (**b**).

The second aim of the research was to investigate the role of flowability sensitivity on food choice. Thus, a choice index for each domain (liquid and solid food) was calculated, for all the participants, as a sum of the choices of the thick options and the hard options, assigning to each one a value of 1, with higher scores reflecting a higher choice of the thick and hard options. The distribution of liquid (a) and solid (b) choice indexes is shown in (Figure 3).

From the distribution of the LCI and SCI, median values were extrapolated to cluster subjects into two groups of preference. Regarding the liquid domain, subjects with an LCI less than the median value (median LCI = 3) were clustered as subjects who preferred the thin version of the proposed foods (thin-food lovers); subjects with an LCI higher than the median value, in contrast, were clustered as subjects who preferred the thick version of the proposed foods (thick-food lovers). In the same way, regarding the solid domain, subjects with an SCI less than the median value (median SCI = 2) were clustered as subjects who preferred the soft version of the proposed foods (soft-food lovers); subjects with an SCI higher than the median value, in contrast, were clustered as subjects who preferred the hard version of the proposed foods (hard-food lovers).

**Figure 4:**
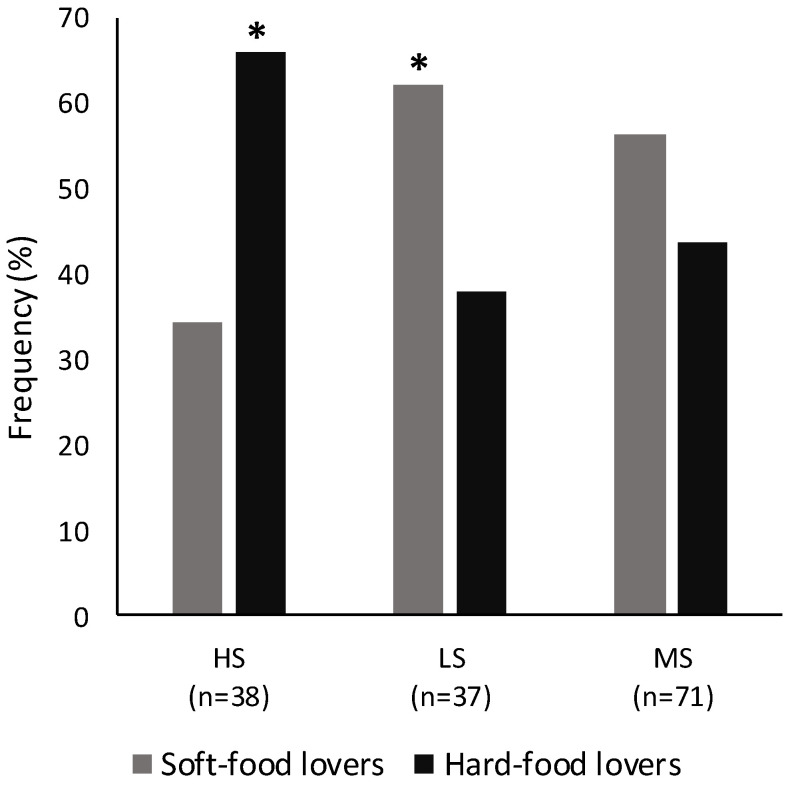
Distribution (%) of soft-food lovers and hard-food lovers in the lowly (LS, *n* = 37), moderately (MS, *n* = 71) and highly (HS, *n* = 38) sensitive groups. The bar marked with a star (*) was significantly different (*p* ≤ 0.05).

The relationship between flowability sensitivity and the individual food choice was tested by running a chi-square test using the three groups of sensitivity and the two groups of preference as variables. Considering the liquid domain, no significant relationships were found (χ^2^ = 0.75, *p* = 0.69). This means that the thin- and thick-food lovers were equally distributed among the three groups of sensitivity. However, the solid choice was strongly affected by the flowability sensitivity (χ^2^ = 6.9, *p* = 0.03) (Figure 4). In particular, highly sensitive subjects were significantly more represented by hard-food lovers, while lowly sensitive subjects were significantly more represented by soft-food lovers. The two solid preference groups were equally distributed in the moderately sensitive group. 

The third aim of this research was to explore the relationship between flowability sensitivity and individual neophobia traits. 

Neophilic (*n* = 71) and neophobic (*n* = 75) subjects did not significantly differ in the flowability sensitivity group distribution (χ^2^ = 3.16; *p* = 0.21). 

It is, however, possible that although their perceptual abilities are not different, their appreciation of different levels of texture stimuli could be. With this assumption, repeated one-way ANOVA was run, using the two neophobia levels as fixed variables, to investigate the differences in food liking within the groups. There were no significant differences among the food liking scores given by neophilic subjects (F_4,280_ = 1.35; *p* = 0.25). Therefore, neophilic subjects equally liked all the tasted samples and did not discriminate between them in terms of liking. In contrast, neophobic subjects’ scores were significantly different among the samples (F_4,296_ = 3.3; *p* = 0.01), with higher scores for intermediate concentrations. 

## 4. Discussion

### 4.1. Rheological Properties: Stress Overshoot

Shear stress was measured as a function of time at the estimated shear rate of the mouth, according to [35]. As expected, by increasing the solid concentration, the maximum shear stress value (stress overshoot, Pa) increased accordingly. The higher the solid concentration, the higher the stress overshoot [43,44,45,46,47]. Stress overshoot data were found useful in modelling the human perception of fluid thickness in the mouth [35]. The stress overshoot better reflects the complexity of the oral evaluation mechanisms related to flowability compared to dynamic viscosity [48]. The trend in the maximum stress overshoot was described with a linear equation (*R*^2^ = 0.98). The linear correlation observed is in accordance with the study conducted by [49].

### 4.2. Sensory Evaluation

Considering both food liking and perceived flowability average scores, significant differences were found among the samples (Table 2).

The effect of familiarity with texture sensations on texture preferences is well documented [50,51,52,53]. Indeed, our results agree with the study conducted by Richardson–Harman et al., [54], who showed how the liking of a range of liquid dairy products is affected by unfamiliar viscosities due to different fat contents. More in general, a familiar texture strongly affects the consumers’ preference, as demonstrated in the study conducted by Kälviäinen and colleagues [55]. The authors demonstrated that consumers’ preferences for candies are explained by their liking of commercial candies with a similar texture.

In the present study, as already explained, the optimal and familiar flowability of cream can be obtained with the third concentration (C3, 36% *w*/*v*, ~400 Pa). The third sample was the most liked one, which means that people (on average) particularly liked the samples with which they are familiar.

Moreover, as already stressed above, familiarity with a well-known sensory property influences the consumers’ sensory perception [56]. In particular, the study conducted by Kim and colleagues [57] showed that Korean consumers are more able to discriminate among green teas than French consumers, who are not familiar with that kind of beverage. Two years later, the same authors demonstrated that even trained panels are affected by familiarity and liking when asked to sensory-describe different samples of tea [58].

Considering the perceived flowability intensity, subjects equally perceived the flowability of the first two samples (C1, 31%; C2, 33% (*w*/*v*)) as well as of the last two (C4, 39%; C5, 43% (*w*/*v*)). The flowability of the intermediate sample (C3, 36% (*w*/*v*)) was perceived as significantly different compared to the perceived flowability of the other samples.

Applying the method proposed by Puleo and colleagues [18,32], and thus considering subjects clustered for different flowability sensitivities, the lowly sensitive subjects evaluated the perceived flowability of the two less concentrated samples with higher scores compared to moderately and highly sensitive subjects. On the contrary, the lowly sensitive subjects evaluated the perceived flowability of the two most concentrated samples with lower scores compared to moderately and highly sensitive subjects. This behaviour confirmed the weak acuity of the lowly sensitive group in contrast with the highly sensitive group, who gave upward scores as the solid concentration increased. The results are absolutely in agreement with previous research [18,32]. Puleo and colleagues clustered people according to their graininess [18] and hardness [32] sensitivity, basing their approach on the correlation between the instrumentally measured parameters and sensory perceptions.

In contrast with the above-cited studies [18,32], no significant effect of age (*p* = 0.297) and gender (*p* = 0.78) on flowability sensitivity was found. Although we had a quite heterogeneous group of subjects (*N* = 176; females = 118, age range = 18–70 years old, median age = 25 years), this last result was in agreement with the study conducted by Steele and colleagues [16], who found no significant relationship between viscosity sensitivity and age and gender.

Considering the role of texture sensitivity in food liking, only a limited number of studies have been conducted in this area of research so far, and all of them have not only used different products but also used different procedures and/or subject criteria [19,59,60,61].

In the present study, by looking at the differences in terms of food liking within the sensitivity groups, the lowly sensitive subjects equally liked all the samples. This result was quite expected, considering that flowability was the only attribute that varied among the samples, and the lowly sensitivity group was not able to perceive the differences in terms of flowability. On the contrary, the highly sensitive subjects differently liked the samples, preferring the middle one (C3, 36% (*w*/*v*)). Moreover, by looking at the differences in terms of food liking between the sensitivity groups, a significant difference was only found regarding the middle sample. In particular, the highly sensitive subjects evaluated this sample with higher scores compared to the lowly and moderately sensitive subjects. Therefore, subjects who were able to successfully discriminate among the different solid concentrations of the samples liked more the sample with the ideal flowability, which, being recognised as optimal and familiar, was more accepted by them.

These results seem to be in contrast with the findings of Kremer and colleagues [59,60], who showed how individuals who differed in texture perception exhibit no clear difference in food-liking scores when evaluating custards and soups differing in creaminess. However, the contrasting results could be due to the fact that the texture sensitivity methods used in their study were not fully validated and the collected research data were scarce because they were measured on a limited number of subjects.

The second aim of this research was to investigate the role of flowability sensitivity on food choice. To discuss the results described above, a direct comparison with other research is not possible, because, to the best of the knowledge, the methodology used in this study has not been adopted by other authors. However, some considerations based on the studies reported in the literature can be done. The individual differences in sensitivity found resulted in influencing the food choice and preference. This last result seems to be in contrast with the findings of Lukasewycz and Mennella [38], who measured the lingual acuity using a modified letter identification task and a forced-choice questionnaire assessed to measure the preferences for foods similar in flavour but different in texture. They involved children and their mothers and concluded that age, but not lingual acuity, influenced food choices.

In our case, it can be noted that in developing the food choice questionnaire, we took into account four levels of flowability. The hard options represented the last considered level, which was not flowing at all. Therefore, we may speculate that the higher the sensitivity, the lower the preference for flowing foods.

Generally speaking, considering the proposed approach to cluster subjects according to their flowability sensitivity, the moderately sensitive group represents the average population and, therefore, reflects the behaviour of the average consumer. The highly and lowly sensitive groups, instead, are, respectively, the right and left tails of the flowability sensitivity distribution, representing outlier consumers. Thus, understanding how texture sensitivity can drive food choices is necessary for food companies intending to develop new, tailored food products for specific consumer targets.

Finally, considering the third aim of this research, no significant relationship was found between flowability sensitivity and individual neophobia.

Food neophobia is considered an adaptive, evolutionary response that prevents the ingestion of poisonous substances more commonly found in fruits and vegetables (i.e., bitter, sour and astringent compounds) [62]. Therefore, it could have been reasonable to hypothesise that neophobic subjects are more sensitive in the sensory perception, also detecting little changes in food properties.

However, the present result is in accordance with the study of Lukasewycz and Mennella [38]. They measured whether lingual tactile acuity—the ability to identify raised alphabetical letters with the tips of their tongues—in children and adults is related to food neophobia. No such relationship was found, which suggests that neophobic subjects are not more sensitive in texture perception. If extended to sensitivity to other stimuli, the present results are also in accordance with the study conducted by Törnwall and colleagues [23], where neophobic and neophilic subjects did not differ in their PROP responsiveness.

In addition, considering that food neophobia primarily reflects the degree of reluctance to consume novel foods, there is considerable evidence that high levels of food neophobia are associated with reduced preference [26,27,63]. In terms of food liking, neophilic subjects in the present study equally liked all the tasted samples and did not discriminate between them in terms of liking. In contrast, neophobic subjects’ scores were significantly different among the samples (F_4,296_ = 3.3; *p* = 0.01), with higher scores for intermediate concentrations.

This last evidence can be explained by the fact that high levels of neophobia reflect a rejection of unfamiliar foods [64]. In addition, subjects having high levels of food neophobia are possibly not only those who are afraid of new foods but also individuals who have little interest in foods [63].

On these reasonable hypotheses, the present results are in accordance with other studies that showed a strong relationship between food neophobia levels and food familiarity [22,42]. Those studies demonstrated that neophobic subjects like unfamiliar food significantly less than neophilic ones.

This last part deserves further consideration. Although no significant relationship was found between flowability sensitivity and food neophobia, the fact that neophobic subjects discriminated between the samples in terms of liking may suggest the existence of a different perception. Thus, since flowability was the only thing that changed among the five chocolate creams, it seems that neophobic subjects were able to perceive those differences in contrast to what was observed with neophilic subjects. Therefore, neophobic subjects seemed to show a higher flowability acuity than neophilic ones. As a final remark, it can be highlighted that the actual product prepared in this study is rather familiar in Italy. Thus, it would be interesting to replicate the study to verify whether the relationship between texture sensitivity and food neophobia would be stronger when using novel and unfamiliar foods.

Finally, the number of subjects involved in this study was relatively small (*n* = 176); therefore, the division of respondents into both flowability sensitivity and food neophobia groups has a more exploratory character.

Further research in this area is needed to establish whether these results are legitimate. In addition, considering that the approach used to measure the flowability sensitivity has been already used in previous studies [18,32], it would be interesting to explore the relationships between sensitivities to different texture attributes measured with this same approach. This topic is currently under investigation.

## 5. Conclusions

This study was conducted to better explore the role of texture sensitivity and food neophobia in food preferences and choices, which are rather dubious. The sensitivity to flowability sensation was investigated using chocolate creams with different levels of flowability. Firstly, instrumental data showed that different solid concentrations result in different levels of stress overshoot and that this index is correlated with the solid concentration by a linear equation. By assuming that sensory flowability scores must correlate with the solid concentration in the same way the stress overshoot index does, subjects were clustered into three groups based on flowability sensitivity, according to the *R*^2^ values and the estimated angular coefficient of the linear equation, derived by the best fit of the data relative to each subject. The flowability sensitivity significantly affected the liking of chocolate creams. Secondly, the flowability sensitivity also affected the solid food choice. In particular, highly sensitive subjects preferred the hard options corresponding to the not-flowing-at-all foods.

Finally, flowability sensitivity was not correlated with the individual level of food neophobia. However, food neophobia affected the liking of chocolate creams, confirming that the reluctance to try unknown foods strongly influences and drives food preferences.

The presented findings confirm that food preferences and choices are influenced by many individual factors, both related to the sensory acuity and psychological traits.

## Figures and Tables

**Table 1:** Texture dichotomies belonging to two texture domains used in the FCQ.

**Liquid Texture Domain**	**Thin Option**	**Thick Option**
	Milk	Yoghurt
	Coffee	Cream of coffee
	Fruit juice	Fruit centrifuge
	Fruit smoothie	Fruit frappé
	Vegetable broth	Vegetable creamed soup
**Solid Texture Domain**	**Soft Option**	**Hard Option**
	Italian ice cream	Ice lolly
	Sandwich bread	Crackers
	Soft chocolate snack	Chocolate bar
	Plum cake	Cookies

**Table 2:** Food liking and perceived flowability scores (mean value ± standard error) given by 176 subjects.

Sample Code	Solid Concentration (% *w*/*v*)	Food Liking (LAM Scale)	Perceived Flowability (gLM Scale)
C1	31	60 ± 1 ^a^	22 ± 1 ^a^
C2	33	60.5 ± 0.9 ^ab^	21 ± 1 ^a^
C3	36	63.5 ± 0.9 ^c^	28 ± 1 ^b^
C4	39	63.1 ± 0.9 ^bc^	40 ± 1 ^c^
C5	43	62.6 ± 0.9 ^abc^	37 ± 2 ^c^

In each column, the values followed by different letters were significantly different (Duncan’s test, *p* ≤ 0.05).

**Table 3:** Quartile distribution of angular coefficients and *R*^2^.

Quartiles	Angular Coefficient	*R* ^2^
1st Quartile	113.0	0.40
Median	176.4	0.67
3rd Quartile	294.8	0.89
Maximum	776.0	0.99
Minimum	1.99	0.001

**Table 4:** Perceived flowability and food liking scores (mean value ± standard error) given by different groups: high-sensitivity (HS) group, low-sensitivity (LS) group and moderate-sensitivity (MS) group.

**Perceived Flowability (gLM Scale)**						
**Groups**	**C1** **31% *w*/*v***	**C2** **33% *w*/*v***	**C3** **36% *w*/*v***	**C4** **39% *w*/*v***	**C5** **43% *w*/*v***	**Significance Within Groups**
LS (*n* = 48)	29 ± 2 ^bA^	26 ± 2 ^bA^	29 ± 2 ^aA^	28 ± 3 ^aA^	23 ± 3 ^aA^	0.12 ^n.s.^
MS (*n* = 83)	20 ± 2 ^aA^	19 ± 2 ^aA^	28 ± 2 ^aB^	36 ± 3 ^bC^	40 ± 2 ^bC^	<0.0001
HS (*n* = 45)	17. ± 3 ^aA^	18 ± 2 ^aA^	25 ± 2 ^aB^	36 ± 2 ^abC^	45 ± 3 ^bC^	<0.0001
Significance between groups	0.003	0.01	0.5^n.s.^	0.03	0.0001	-
**Food Liking (LAM scale)**						
**Groups**	**C1** **31% *w*/*v***	**C2** **33% *w*/*v***	**C3** **36% *w*/*v***	**C4** **39% *w*/*v***	**C5** **43% *w*/*v***	**Significance Within Groups**
LS (*n* = 48)	60 ± 2 ^aA^	62 ± 2 ^aA^	61 ± 2 ^aA^	63 ± 2 ^aA^	60 ± 2 ^aA^	0.4 ^n.s.^
MS (*n* = 83)	61 ± 2 ^aAB^	60 ± 1 ^aA^	63 ± 1 ^abAB^	62 ± 1 ^aAB^	65 ± 1 ^aB^	0.02
HS (*n* = 45)	59 ± 2 ^aA^	60 ± 2 ^aA^	67 ± 2 ^bC^	66 ± 2 ^aBC^	62 ± 2 ^aAB^	0.003
Significance between groups	0.89 ^n.s.^	0.62 ^n.s.^	0.04	0.24 ^n.s.^	0.10 ^n.s.^	-

For each line (uppercase A–C) and each column (lowercase a,b), different letters significantly correspond with different values (Duncan’s test, *p* ≤ 0.05). n.s.: not significant (*p* > 0.05).

## Data Availability

The data presented in this study are available on request from the corresponding author.

## Supplementary Materials

The following are available online at https://www.mdpi.com/article/10.3390/foods10051024/s1, Table S1: Ranking test results: multiple pairwise comparisons using Nemenyi’s procedure/two-tailed test.
