# Supplementary material for: Oral Sensitivity to Flowability and Food Neophobia Drive Food Preferences and Choice
Source: Foods. 2021 May 8;10(5):1024. doi: 10.3390/foods10051024 (PMC8150315; doi:10.3390/foods10051024)
Supplement: Supplementary file 1 [file foods-10-01024-s001.zip › foods-1182418-SI.pdf]

Supplementary Material

Table S1. Ranking test results: multiple pairwise comparisons using Nemenyi's procedure/ Two-tailed test.

**Solid concentration (% *w*/*v*)**	**Sum of ranks**	**Mean of ranks**	**Groups†**				
54	39	1.219	A				
48	63	1.969	A				
43*	94	2.938	A	B			
39*	128	4.000		B	C		
36*	163	5.094			C	D	
33*	191	5.969				D	E
31*	224	7.000					E
29	250	7.813					E

† $p < 0.0001$; *Samples selected for the consumer test.
